# Iatrogenic cushing's syndrome in children following nasal steroid

**DOI:** 10.11604/pamj.2014.17.237.3332

**Published:** 2014-03-28

**Authors:** Isaac Oludare Oluwayemi, Abiola Olufunmilayo Oduwole, Elizabeth Oyenusi, Alphonsus Ndidi Onyiriuka, Muhammad Abdullahi, Olubunmi Benedicta Fakeye-Udeogu, Chidozie Jude Achonwa, Moustapha Kouyate

**Affiliations:** 1 Paediatric Endocrinology Training Centre for West Africa, Lagos University Teaching Hospital, Idi-araba, Lagos, Nigeria

**Keywords:** Iatrogenic, cushing's syndrome, nasal steroid

## Abstract

Cushing syndrome is a hormonal disorder caused by prolonged exposure of body tissue to cortisol. We report two cases of iatrogenic Cushing's syndrome in two Nigerian children following intranasal administration of aristobed-N (Betamethasone + Neomycin) given at a private hospital where the children presented with feature of adenoidal hypertrophy. Two months into treatment children were noticed to have developed clinical and laboratory features of iatrogenic Cushing's syndrome with critical adrenal suppression. Serum cortisol (at presentation): 1^st^ patient: 12nmol/L (reference range 240-618), 2^nd^ Patient: 1.69nmol/L. Serum cortisol (3 months after weaning off steroid): 343.27 nmol/L (within normal range for the first patient; second patient newly presented and has just begun steroid weaning off process. The serum cortisol level one month into weaninig off process was 128 nmol/L). Unsupervised topical steroid administration in children can cause adrenal suppression with clinical features of Cushing's syndrome.

## Introduction

Cushing syndrome is a hormonal disorder caused by prolonged exposure of body tissue to cortisol. Cushing's syndrome is relatively rare and most commonly affect adult between the ages of 20 and 50 years [1, 2]. It has an incidence of 2-5 new cases /million people/year and 10% of these new cases occur in children [1]. We report two children who developed clinical and laboratory features of iatrogenic Cushing's syndrome following unsupervised administration of intranasal steroid.

## Patients and case report

C.W. a 19 month old boy was referred to the Paediatric endocrinology clinic of Lagos University Teaching Hospital with a 2 month history of excessive weight gain and unusual pubic hair growth. Child was seen at a private hospital 3 months earlier where he presented with history recurrent nasal discharge and snoring while sleeping. He was placed on aristobet-N (Betamethasone + Neomycin) nasal drop, one drop per nostril 3 times daily for three months. At the time of presentation, child has taken six bottles (30mg) of aristobet-N. However 2 months into using the drugs parents discovered that child was gaining weight inappropriately and his face became round and eye lids very full (previous clothes became too tight for him). Child was also noticed to have increased pubic hair growth (though child was said to have been very hairy since birth and father also hairy). His birth weight was 3.2kg (25^th^percentile)

**Figure 1:**
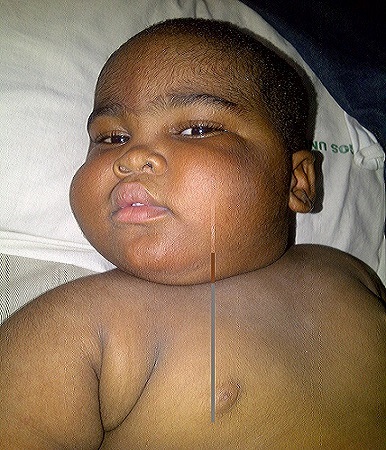
Cushingoid facies of the 1st affected child

At presentation, child looks obese, had round moon-like face (Figure 1) excessive hair on the forehead, back and on the pubis. No axillary hair. Testicular volume was 1ml (prepubertal). Weight was 17.5kg (>95^th^ percentile) and within one week it increased to 18.0kg; height was 87.5cm (75^th^ percentile); BMI= 22.9 kg/m^2^; OFC= 48.5cm (50^th^ percentile); MAC = 19cm

Intranasal Betamethasone was gradually weaned off over 3 weeks and child progressively lost 1.5 kg during this period (weight was 16.5kg by the time Betamethasone was finally stopped). Based on the history, clinical features and markedly reduced serum cortisol & ACTH an assessment of iatrogenic Cushing's syndrome with critical adrenal suppression was made; parents were counseled on need to report to the hospital any time child is sick. Two weeks after discontinuation of Betamethasone child had tonsilopharyngitis and was given oral hydrocortisone 12mg/m^2^ /day (5mg tds) for 3 day and amoxyl-clavulanate 356mg bd for 10 days. A repeat serum cortisol in 3 months after weaning off steroid was within normal limit (343.27nmol/L). Child is presently doing very well.

**Figure 2:**
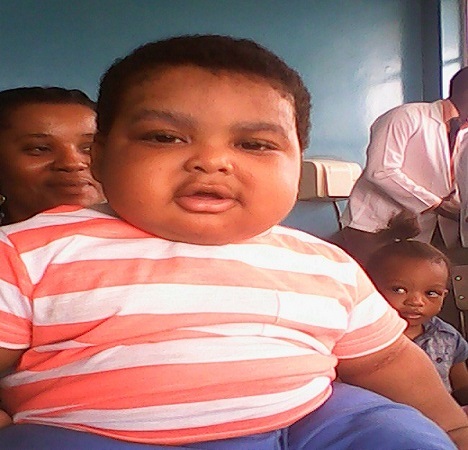
Cushingoid facies of the 2nd affected child

The second patient E.N. presented 4 months after the first patient presented and amazingly from the same private hospital! E.N. (Figure 2) is a 9 month old male infant who presented with 7 week history of excessive weight gain. He has presented 2 months earlier in a private hospital with 3 month history of noisy breathing for which a diagnosis of adenoidal hypertrophy was made and he was given aristobed-N (Betamethasone + Neomycin) 2 drops per nostril tds. The nasal drop was administered by both mother and relatives and he finished 3 bottles per week! (about 21 bottles in 7 weeks!). His birth weight was 3.6kg; at 6 weeks 5.7kg; and at 9 month 16.2kg. When child was observed to be gaining weight, he reported back at the private hospital and child was referred to Paediatric endocrinology unit with serum cortisol level of 1.69nmol/L (reference range 240-618). Essential finding at presentation were an obese infant, full round face, length 62cm (< 5^th^ percentile), BMI 36.7kg/m^2^ (>95^th^ percentile); BP 100/60mmHg. Other systems were essentially normal.

An assessment of iatrogenic Cushing's syndrome was made and was weaned off betamethasone and commenced on oral hydrocortisone 6mg/m^2^ / day. The following are the investigation result of the first patient: Abdominal USS: Normal study; Random blood sugar (RBS): 5.6mmol/L (n); serum cortisol: 12nmol/L (normal range: 240-618). Three months after weaning off steroid the serum cortisol rose to 343.27 nmol/L) Serum ACTH: 0.2pmol/L (1.6-13.9)’; bone age: compatible with 18 months, reduced bone density; serum calcium total: 2.50 mmol/L (2.25-2.75); calcium corrected: 2.45 mmol/L (2.25-2.75); serum Albumin: 42 g/L (35-50); phosphate: 1.52 mmol/L (0.81-2.07)

The second patient had the following investigation results: Serum Cortisol: 1.69nmol/L (normal range: 240-618). One month after commencement of weaning off steroid, the serum cortisol rose to 128nmol/L; bone age was compatible with child's age

## Discussion

**Figure 3:**
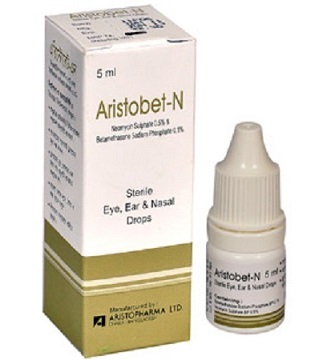
Picture of intranasal steroid administered

Topical steroid are commonly used in treatment of allergic rhinitis and asthma. They are commonly considered safer compared to systemic steroids since it is generally believed that there is limited absorption topically. Cushing's syndrome following the use of topical steroids is rare [3] but where administration is not well supervised quite a large amount could be administered inadvertently especially when dealing with uncooperative children [4, 5]. The patient in this presentation is likely to have taken over dosage of the intranasal steroid considering the fact that he was given 6 bottles in 3 months. Ideally a bottle of 5mls of betamethasone contain 150 drops according to the manufacturer [6] and at one drop per nostril t.d.s a bottle should have lasted 25 days. Each ml of Aristobet-N (Figure 3) contains Betamethasone Sodium Phosphate 1mg and Neomycin Sulphate BP 5mg. Our first patient however was given more than twice the prescribed dose probably inadvertently and that for 3 months. While the second patient had 21 bottles (100mg) of betamethasone in 7 weeks! This could explain features of Cushing syndrome seen in these patients. It is important that children placed on topical or systemic steroid are followed up closely and prescription reviewed regularly to prevent avoidable and costly iatrogenic Cushing syndrome. It is worthy of note that this might just be a tip of the ice barge representation of so many children who might not be lucky enough to be seen by very few Paediatric endocrinologists especially in developing countries.

## Conclusion

Topical steroid administrations in children have grave consequences on the health of children and should be prescribed with great caution and only for a very short period. Children with features reminiscent of Cushing's syndrome should be referred promptly to Paediatric endocrinologist for management.
